# Glacier fluctuations in the northern Patagonian Andes (44°S) imply wind-modulated interhemispheric in-phase climate shifts during Termination 1

**DOI:** 10.1038/s41598-022-14921-4

**Published:** 2022-06-27

**Authors:** Rodrigo L. Soteres, Esteban A. Sagredo, Michael R. Kaplan, Mateo A. Martini, Patricio I. Moreno, Scott A. Reynhout, Roseanne Schwartz, Joerg M. Schaefer

**Affiliations:** 1 Instituto de Geografía, Pontificia Universidad Católica de Chile, Campus San Joaquín, Avda. Vicuña Mackenna 4860, Macul, Santiago, Chile; 2 Millennium Nucleus Paleoclimate, ANID-Millennium Science Initiative, Santiago, Chile; 3 Estación Patagonia de Investigaciones Interdisciplinarias UC, Pontificia Universidad Católica de Chile, Santiago, Chile; 4 Lamont-Doherty Earth Observatory of Columbia University, Palisades, NY USA; 5 Centro de Investigaciones en Ciencias de La Tierra (CONICET-UNC), Facultad de Ciencias Exactas, Físicas y Naturales, Universidad Nacional de Córdoba, Córdoba, Argentina; 6 Center for Climate Research and Resilience, Universidad de Chile, Santiago, Chile; 7 Institute of Ecology and Biodiversity, Universidad de Chile, Santiago, Chile; 8 Departamento de Ciencias Ecológicas, Universidad de Chile, Santiago, Chile; 9 Departamento de Geología, Universidad de Chile, Santiago, Chile; 10 Department of Earth and Environmental Sciences, Columbia University, New York, NY USA

**Keywords:** Geomorphology, Palaeoclimate

## Abstract

The Last Glacial Termination (T1) featured major changes in global circulation systems that led to a shift from glacial to interglacial climate. While polar ice cores attest to an antiphased thermal pattern at millennial timescales, recent well-dated moraine records from both hemispheres suggest in-phase fluctuations in glaciers through T1, which is inconsistent with the bipolar see-saw paradigm. Here, we present a glacier chronology based on 30 new ^10^Be surface exposure ages from well-preserved moraines in the Lago Palena/General Vintter basin in northern Patagonia (~ 44°S). We find that the main glacier lobe underwent profound retreat after 19.7 ± 0.7 ka. This recessional trend led to the individualization of the Cerro Riñón glacier by ~ 16.3 ka, which underwent minor readvances at 15.9 ± 0.5 ka during Heinrich Stadial 1, during the Antarctic Cold Reversal with successive maxima at 13.5 ± 0.4, 13.1 ± 0.4, and 13.1 ± 0.5 ka, and a minor culmination at 12.5 ± 0.4 ka during Younger Dryas time. We conclude that fluctuations of Patagonian glaciers during T1 were controlled primarily by climate anomalies brought by shifts in the Southern Westerly Winds (SWW) locus. We posit that the global covariation of mountain glaciers during T1 was linked to variations in atmospheric CO_2_ (atmCO_2_) promoted by the interplay of the SWW-Southern Ocean system at millennial timescales.

## Introduction

The Last Glacial Termination (T1; ~ 18–11.7 ka) represents the largest, most abrupt climate change of the last glacial-interglacial cycle^1^. T1 featured the collapse of continental-scale ice sheets, a persistent rise in both sea level and the concentration of atmospheric greenhouse gases, along with a millennial-scale sequence of climate changes that culminated with the onset of the current interglacial. Ice core data reveal a step-like pattern of increasing atmospheric temperatures featuring a synchronous but antiphased trend at millennial timescale between the polar hemispheres^2,3^. Recently developed precise mountain glacier chronologies in the mid-to-high latitudes of both hemispheres^4–7^ (see Supplementary material S1), however, are starting to reveal in-phase behavior, pointing to a globally consistent mountain glacier response to climate changes during T1. This pattern challenges the expected antiphased or out-of-phase response predicted by the bipolar seesaw paradigm. If replicated by detailed and precise glacial chronologies, this mismatch could reveal key insights about interhemispheric climate links mediated by some atmospheric component operative during glacial terminations.

Recently, Denton et al.^8^ proposed that changes in the Southern Westerly Winds (SWW) are the critical missing link relating insolation, atmospheric greenhouse gas concentrations, atmospheric and ocean circulation, and glacier response at millennial timescale during glacial maxima and terminations. By way of interaction with the Southern Ocean (SO), the SWW establish a coupled system that drives global ocean circulation, the atmospheric concentration of greenhouse gases, and high-latitude ocean productivity by enhancing upwelling of CO_2_-enriched and high-nutrient deep waters^9^. The efficacy of the SWW-SO coupled system is dependent upon the wind stress imparted by the SWW on the surface of the SO south of the Drake Passage (> 55° S)^10^. Hence, deciphering the geographical position and strength of the locus of the SWW are crucial for assessing global climate change during ice age terminations.

The SWW are the sole driver of precipitation to the Pacific and Andean divide sectors of northern Patagonia (40°–44° S)^11^. This region-specific response to incident atmospheric flow allows reconstructing past SWW behavior based on hydrologic balance variations preserved in stratigraphic and geomorphologic records from the Chilean Lake District and Chilotan archipelago (40–44° S). When analyzed in conjunction with similar records from southern Patagonia (50°-54°S), latitudinal shifts and intensity variations in the SWW at millennial-scale during the Last Glacial Maximum (LGM; ~ 35–18 ka) and T1^12,13^ can be identified. However, few studies in northern Patagonia have examined in any detail glacier fluctuations following the onset of T1^14,15^, limiting our understanding about the response of middle latitude austral glaciers and paleoclimate patterns and processes at continental, hemispheric, and global scales.

**Figure 1:**
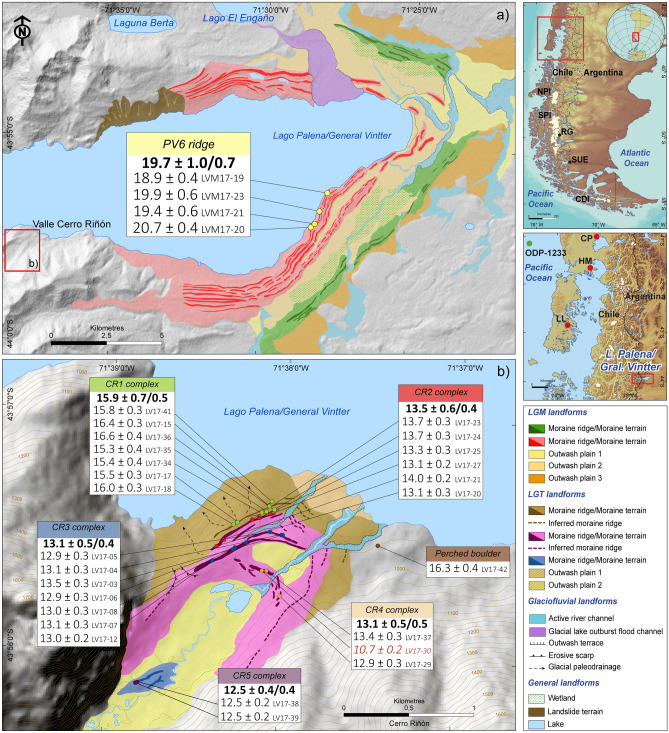
Glacial geomorphology and chronology of the (**a**) Lago Palena/General Vintter ice lobe with ^10^Be ages (n = 4) obtained from the innermost moraine ridge (PV6) and (**b**) the Cerro Riñón valley glacier with ^10^Be ages (n = 26) obtained from CR1-CR5 moraine complexes and the additional perched boulder. On top of the boxes the mean moraine age is acompanied by 1 standard deviation (σ) and the Standard Error of the Mean (SEM) including a 3% propagated production rate error^20^ (Table 1). Individual ages are presented along with internal uncertainty and sample ID. One age in red italics is considered an outlier. Inset maps with the location of sites mentioned in the text. Black dots correspond with glacial chronologies from RG: Río Guanaco; SUE: Seno Última Esperanza. Red dots correspond to paleovegetation reconstruction. LL: Lago Lepué. CP: Canal de la Puntilla. HM: Huelmo mire. Green dot is the location of ODP1233 sediments core. White outlines represent Northern (NPI) and Southern (SPI) Patagonian Icefields and Cordillera Darwin Icefield (CDI). This figure was created on ESRI ArcGIS v10.4 software (www.esri.com).

Mountain glaciers are sensitive to and provide a direct physical link to changing atmospheric conditions. For land-terminating glaciers in particular, moraines distal to present ice limits are unambiguous recorders that past climate changed. Thus, the anatomy of glacier fluctuations during T1 in the mid-latitude Andes not only offers empirical constraints on the interhemispheric synchrony of mountain glacier behavior, but also a means to examine the evolution of the SWW and associated paleoclimates. Building on prior mapping^16^, we present a glacial geomorphologic map and a ^10^Be geochronology of the inner Lago Palena/General Vintter (LPGV) basin, centered at ~ 43.9° S; ~ 71.5° W in northern Patagonia, to examine the timing and structure of glacier fluctuations during T1. This basin was covered by an eastward-flowing outlet lobe of the Patagonian Ice Sheet that was fed through several coalescing valleys during the LGM (Fig. 1)^17^. The foundation of our chronology comes from one of these tributary valleys located ~ 15 km from the southern shore of the lake, at the foot of Cerro Riñón (~ 1790 m asl). The Cerro Riñón valley (informal name) is carved into the North Patagonian batholith^18^, which was the source of the abundant glacially-transported granitoid boulders found on the local landscape.

## Results

Previous studies delineated multiple moraines alongside the eastern half of the LPGV, which have been tentatively assigned to the LGM^15–17,19^. Our geomorphological map allows identification of at least six well-preserved, closely spaced arcuate moraine ridges, the innermost of which we name PV6 (Fig. 1). We obtained four ^10^Be samples from boulders atop PV6, which yielded ages between 20.7 ± 0.4 and 18.9 ± 0.4 ka, with a mean of 19.7 ± 0.7 ka (Fig. 1, Table 1), calculated using the Patagonian regional production rate^20^ and the time-dependent Lal/Stone scaling scheme (Lm; see “Methods”)^21,22^. Upstream from the PV moraines, within the Cerro Riñón tributary valley, we distinguish five well-preserved moraine groups (CR1 to CR5 from the outermost to the innermost). A single ^10^Be sample from a perched erratic boulder resting over polished bedrock outboard of the CR1 moraines, and ~ 30 m above the modern lake surface, affords an age of 16.3 ± 0.4 ka. We interpret this date as a minimum-limiting age for local ice evacuation. The CR1 moraines lie ~ 300 m south from the lake shore and comprise a ~ 500 m long and ~ 10 m high main ridge connected to several minor ridges (Supplementary Fig. S2). We obtained seven ^10^Be samples that range from 16.6 ± 0.4 to 15.3 ± 0.4 ka, with a mean of 15.9 ± 0.5 ka. Directly inside CR1 are two ridges that form the CR2 moraines, clearly distinguishable from CR1 by their larger sizes (~ 700 m long and ~ 20 m high) and sharper appearance (Supplementary Fig. S2). Six ^10^Be samples from the largest and most continuous ridge yielded ages between 14.0 ± 0.2 to 13.1 ± 0.2 ka, with a mean of 13.5 ± 0.4 ka. Immediately inboard, separated by a meltwater channel, the CR3 moraine represents the most continuous (~ 1000 m long) and prominent (~ 30 m high) ice-marginal feature of the area. We obtained seven ^10^Be samples that range in age from 13.5 ± 0.3 to 12.9 ± 0.3 ka, with a mean of 13.1 ± 0.4 ka. Approximately 200 m upstream from CR3, separated by an outwash plain, a group of several discontinuous ridges covered by dense vegetation form the CR4 moraine (Supplementary Fig. S2). Two samples from the outermost moraine ridge of CR4 provide ages of 13.5 ± 0.3 and 12.9 ± 0.3, with a mean of 13.1 ± 0.5 ka (the additional sample LV17-30 (10.6 ± 0.2 ka) was excluded as an outlier^23^) (Fig. 1, Table 1). CR5 comprises the innermost ice-marginal features, located ~ 500 m upstream from CR4, and consists of a ~ 400 m long group of latero-frontal moraine ridge fragments elevated ~ 20 m above the floor of the most extensive outwash plain in this valley. Two ^10^Be samples collected from the outermost ridge yielded statistically identical ages of 12.5 ± 0.2 ka.

**Table 1:** ^10^Be ages from the Lago Palena/General Vintter area calculated using the the non-time-dependent Lal/Stone scaling scheme (St;^21,22^) time-dependent Lal/Stone scaling (Lm;^21,22^ and Lifton et al. scaling (LSDn;^64^).

	Id Sample	Age; St (ka)	Age; Lm (ka)	Age; LSDn (ka)
PV6	LVM17-19	19,210 ± 400	18,860 ± 390	19,050 ± 400
	LVM17-20	21,150 ± 400	20,660 ± 390	20,790 ± 390
	LVM17-21	19,760 ± 570	19,370 ± 560	19,530 ± 560
	LVM17-23	20,280 ± 580	19,860 ± 570	20,000 ± 570
	Mean ± σ/SEM	20,100 ± 1030/730	19,690 ± 970/700	19,840 ± 950/700
CR1	LV17-15	16,620 ± 310	16,440 ± 310	16,640 ± 310
	LV17-17	15,640 ± 330	15,510 ± 330	15,690 ± 330
	LV17-18	16,200 ± 310	16,040 ± 310	16,220 ± 310
	LV17-34	15,550 ± 440	15,420 ± 440	15,630 ± 450
	LV17-35	15,380 ± 350	15,260 ± 350	15,470 ± 360
	LV17-36	16,750 ± 440	16,560 ± 440	16,770 ± 440
	LV17-41	15,980 ± 310	15,830 ± 300	16,040 ± 310
	Mean ± σ/SEM	16,020 ± 720/520	15,860 ± 690/510	16,070 ± 700/520
CR2	LV17-20	13,000 ± 310	13,070 ± 310	13,270 ± 320
	LV17-21	14,000 ± 240	13,980 ± 240	14,170 ± 240
	LV17-23	13,660 ± 260	13,670 ± 260	13,880 ± 260
	LV17-24	13,710 ± 320	13,720 ± 320	13,930 ± 320
	LV17-25	13,210 ± 250	13,260 ± 260	13,500 ± 260
	LV17-27	13,060 ± 250	13,120 ± 250	13,370 ± 250
	Mean ± σ/SEM	13,440 ± 570/430	13,470 ± 550/430	13,690 ± 550/440
CR3	LV17-03	13,500 ± 260	13,530 ± 260	13,760 ± 260
	LV17-04	13,070 ± 290	13,130 ± 290	13,370 ± 300
	LV17-05	12,830 ± 330	12,910 ± 340	13,150 ± 340
	LV17-06	12,780 ± 250	12,870 ± 260	13,090 ± 260
	LV17-07	13,070 ± 290	13,130 ± 290	13,330 ± 300
	LV17-08	12,910 ± 310	12,990 ± 310	13,180 ± 310
	LV17-12	12,880 ± 240	12,970 ± 240	13,160 ± 250
	Mean ± σ/SEM	13,010 ± 460/400	13,070 ± 450/400	13,290 ± 460/410
CR4	LV17-29	12,800 ± 260	12,890 ± 270	13,110 ± 270
	LV17-30*	10,480 ± 240	10,720 ± 250	11,010 ± 250
	LV17-37	13,330 ± 270	13,360 ± 270	13,590 ± 270
	Mean ± σ/SEM	13,060 ± 540/470	13,130 ± 520/460	13,350 ± 520/470
CR 5	LV17-38	12,340 ± 240	12,460 ± 240	12,700 ± 240
	LV17-39	12,420 ± 230	12,540 ± 250	12,770 ± 240
	Mean ± σ/SEM	12,380 ± 380/370	12,500 ± 380/380	12,730 ± 390/380
Perched boulder	LV17-42	16,420 ± 430	16,250 ± 420	16,470 ± 430

Mean moraine ages are shown in bold with 1 outlier excluded (*), and are accompanied by the uncertainty (1 standard deviation [σ]/Standard Error of the Mean [SEM]) considering the propagated error (3%) of the production rate^20^ for both.

## Discussion

Glacial geologic mapping and thirty new ^10^Be ages (1 outlier) constitute the basis of a moraine chronology for the LPGV basin that documents in detail the sequence of glacier/paleoclimatic events during T1 in northern Patagonia. Our data indicate that the retreat of the LPGV glacier lobe from the PV6 moraine at 19.7 ± 0.7 ka likely initiated the present lake, and the ice front did not subsequently re-advance out of its current basin. Considering the dispersion of the PV6 ages, we interpret this date as a maximum-limiting age for presumed large-scale glacier withdrawal during T1. The ice front then retreated more than 40% of its LGM length, prompting the detachment of the Cerro Riñón glacier shortly after 16.3 ± 0.4 ka. After this event, a moraine-building event of this tributary glacier culminated with the deposition of the CR1 moraine at 15.9 ± 0.5 ka. Subsequent advances or standstills deposited in quick succession within the error margin of the dating the CR2 to CR4 moraines between 13.5 ± 0.4 and 13.1 ± 0.4 ka. Cerro Riñón glacier then underwent net recession, only interrupted by a stillstand that constructed CR5 at 12.5 ± 0.4 ka. No ice marginal features are evident up valley, suggesting profound glacier retreat to the headwalls after ~ 12.5 ka. The chronology for the Cerro Riñón glacier informs on the timing and structure of glacier fluctuations throughout the entirety of the T1 chron within a single basin in northern Patagonia, and constrains four moraine deposition phases of similar magnitude (i.e., CR1-CR4) that culminated at ~ 15.9 ka during Heinrich Stadial 1 (HS1: ~ 17.8–14.7 ka) and between ~ 13.5–13.1 ka within the Antarctic Cold Reversal (ACR: ~ 14.7–12.7 ka), followed by an additional advance or stillstand of minor extent (i.e., CR5) at ~ 12.5 ka that is coeval with the Younger Dryas (YD: ~ 12.6–11.5 ka).

The Cerro Riñón glacier expanded and achieved its maximum extent during T1 by ~ 15.9 ka. This result differs from the majority of moraine-based records from southern South America dated so far, which document an apparent sustained and large-scale glacier recession during HS1. Notable exceptions are moraines of similar age observed along the eastern flank of the southern Patagonian Andes, such as the ^10^Be dated Cerro Pintado moraine at Río Guanaco (~ 50°S)^24^ and the ^14^C-constrained Lago Pinto moraine at Última Esperanza (~ 50°S)^25^. Subsequent to HS1, glacier activity in the Cerro Riñón valley represents the northernmost direct evidence for glacier advances during the ACR and gradual retreat during the YD in Patagonia, expanding the known geographical footprint of these glacier/paleoclimate events from 47.5^o^ S to 43.9^o^ S^26–29^. The recent interpretation of an ACR maximum based on geomorphic analysis and lake sedimentary record of former Rosselot glacier^30^, ~ 45 km directly west of Cerro Riñón, is consistent with our results. Considering that Patagonian ice-marginal features formed during the early phases of T1 are often closely spaced, these findings indicate that equilibrium line altitude changes (ELA) at ~ 15.9 ka and during the ACR (CR1-CR4) were similar in magnitude and were followed by a net ELA rise during the YD (CR5), accounting for modest glacier advances or standstills well within ACR limits. At similar latitude, but ~ 9000 km west of LPGV, several moraine records in New Zealand reflect synchronous glacier behavior: massive recession from the LGM limits was punctuated by deposition of the Prospect Hill moraines at ~ 15.9 ka in the Rakaia valley^31^, which was followed by several ACR advances, and then subsequent YD recession was interrupted by minor stillstands in multiple valleys of the Southern Alps^31–36^ (recalculated, see Supplementary material Table S2-S5). We propose that trans-Pacific glaciers fluctuated in unison at millennial timescales through T1, in response to zonally synchronous changes in the SWW and associated climate anomalies^26,33^.

**Figure 2:**
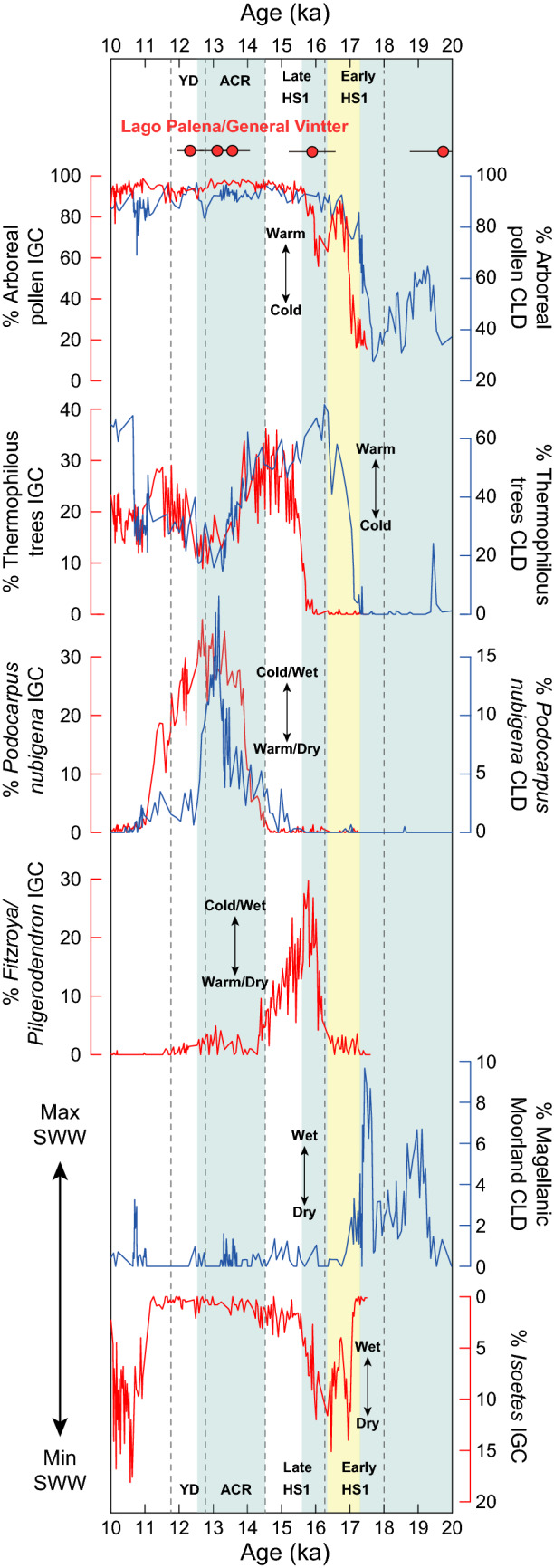
Glacier chronology of the Lago Palena/General Vintter basin and selected species from Canal de la Puntilla and Huelmo mire from the Chilean Lake District (CLD)^12^ and Lago Lepué pollen record from Isla Grande de Chiloé (IGC)^40^. Blue bars highlight pollen-based cold/wet intervals and yellow bars denote pollen-based warm/dry periods within T1.

**Figure 3:**
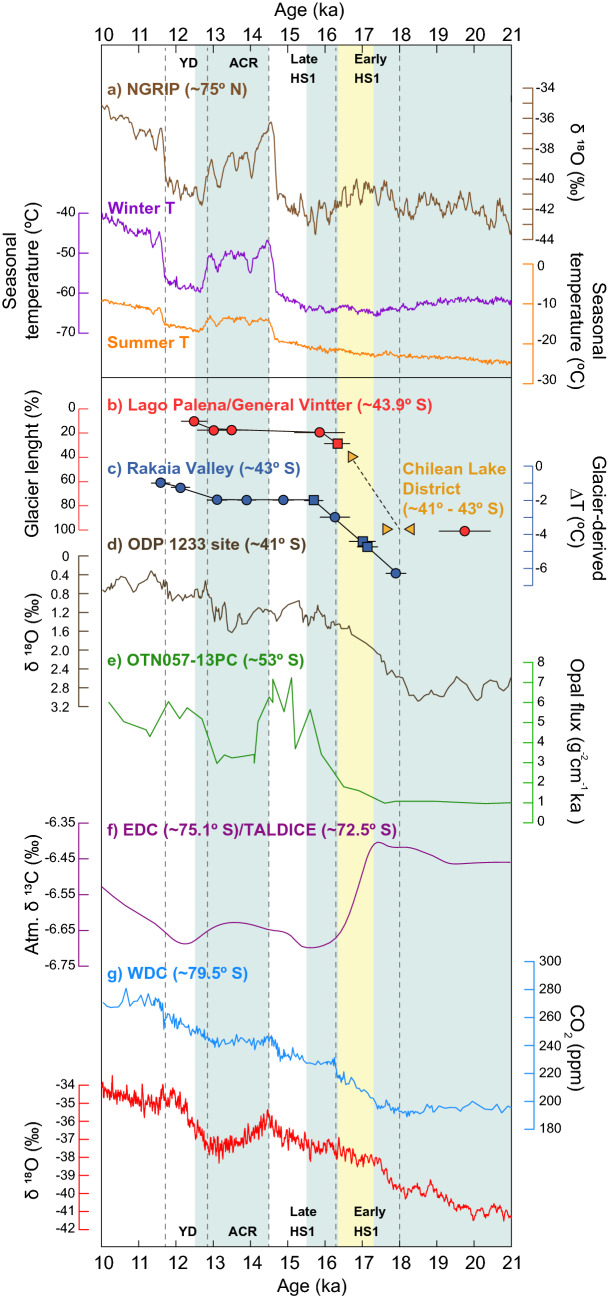
Paleoclimate proxies spanning T1. (**a**) Records from the North Greenland Ice Core Project (NGRIP): δ^18^O^3^; winter temperatures in purple^57^; summer temperatures in yellow^57^. (**b**) Glacier length of the Lago Palena/General Vintter ice lobe (this study). Yellow triangles are bracketing radiocarbon ages for the final glacial advance (100% length) of the LGM in the Chilean Lake District^14^. (**c**) Glacier-derived temperatures from Rakaia Valley, New Zealand^31,32^. (**d**) Planktonic δ^18^O from marine core ODP-1233^28^. (**e**) Opal flux from marine core TN057-13PC^9^. (**f**) Integrated δ^13^C from Talos Dome (TALDICE) and EPICA Dome C (EDC)^43^. (**g**) Records from the central West Antarctica Ice Core (WAIS): CO_2_ in light blue and δ^18^O in red^42^. Blue bars highlight cold/wet intervals and yellow bars denote warm/dry periods inferred from palynological analyses.

We assess the representativeness of the Cerro Riñón glacier record with proxy evidence from lake sediment cores obtained in the Pacific sector of northern Patagonia, which afford valuable information for tracking the regional evolution of the SWW through the LGM and T1^37–39^. Pollen records from the Canal de la Puntilla and Huelmo mire in the Chilean Lake District^12^ and Lago Lepué in Isla Grande de Chiloé^40^ indicate a treeline ~ 1000 m lower than present and presence of Magellanic Moorland communities in the lowlands during the LGM, attesting to cold and hyperhumid conditions brought by a northward shift of the SWW (Fig. 2). This was followed by rapid arboreal expansion, disappearance of Magellanic Moorland driven by deglacial warming and a southward shift of the SWW starting at ~ 17.8 ka. The interval between ~ 17.8–16.4 ka features a low lake-level stand, with peak abundance of the littoral macrophyte *Isoetes* (Fig. 2), signaling low SWW influence during the initial phase of T1. Discrete increases in precipitation occurred at ~ 16.4 ka and ~ 14.7 ka, as indicated by conspicuous increases in cold-tolerant hygrophilous conifers (*Fitzroya/Pilgerodendron* and *Podocarpus nubigena*, respectively). These changes suggest successive incremental increases for SWW influence in northern Patagonia. The earliest of these increases on the wind belt influence lasted until ~ 15.9 ka, as the deglacial warming trend resumed and crossed a critical threshold that favored the diversification and densification of other thermophilous rainforest trees and vines at the expense of *Fitzroya/Pilgerodendron*. The youngest increase in conifers (*Podocarpus nubigena*) during T1 took place between ~ 14.7–12.6 ka, and was followed by enhanced fire activity, a lake-level fall, and decline in conifers after ~ 12.6 ka, which suggest a decrease in precipitation related to a southward shift of the SWW. Wind-driven hydroclimate changes toward cold/wet conditions closely track glacier advances in the LPGV area. We therefore conclude that our record shows a coherent cryospheric response to significant SWW-modulated climate fluctuations during T1. In addition, we note that the reservoir-age corrected paleoclimate records from marine core ODP1233^41^, collected offshore from northwestern Patagonia (Fig. 3), show a pattern consistent with our terrestrial-based chronology of climate change through T1.

Widespread glacier withdrawal in Patagonia^42^ was contemporaneous with distinct atmospheric CO_2_ (atmCO_2_) changes recorded at the beginning and end of T1, between ~ 18.1–16.3 ka and ~ 13–11.7 ka in the WAIS ice core^43^, enhanced ocean ventilation inferred from a rise in opal flux recorded in the SO^9^, and reduced δ^13^C composition of atmCO_2_ preserved in the EPICA Dome C (EDC) and the Talos Dome (TALDICE) ice cores from Antarctica^44^ (Fig. 3). Glacier advances or stillstands that stalled profound ice recession in northern Patagonia coincided with centennial-scale halts in the rising atmCO_2_ trend followed by atmCO_2_ plateaus between ~ 16.3–14.8 ka and ~ 14.8–13.0 ka^43^, concomitant with decreased deep SO water ventilation^9^ and minimum δ^13^C composition of atmCO_2_ from Antarctic ice cores^44^ (Fig. 3).

Our collation of mid- and high-latitude paleoclimate data from the Southern Hemisphere suggests that variations in the strength and/or position of the SWW links hydroclimate changes and glacier mass balance variations in the temperate regions of South America and New Zealand. This trans-Pacific atmospheric circulation pattern can explain the observed terrestrial changes, along with simultaneous upwelling and ventilation of deep waters in the SO. Overall, we note that negative mass balance driving glacier recession from the LGM terminus in the LPGV basin was contemporaneous with negative anomalies in SWW influence in northwestern Patagonia^40^, along with a sustained increase in atmCO_2_^43^ that coincided with invigorated SO upwelling^9^. Collectively, these data indicate a poleward shift of the SWW early during T1^9^. Subsequently, a moraine-building event of the Cerro Riñón glacier indicates a positive mass balance episode culminating at ~ 15.9 ka that was concomitant with positive anomalies in SWW at ~ 44° S^40^, with a pause in the rising trend of atmCO_2_ rapidly starting at 16.3 ka^43^, and subdued increase in SO upwelling^9^. We interpret these correspondences as a simultaneous widening of the SWW belt during the HS1. This was followed by enhanced positive glacier mass balance accounting for multiple ice readvances between ~ 13.5 and 13.1 ka coeval with positive anomalies of the SWW in northwestern Patagonia^40^, and a stall in the rising of atmCO_2_ trend^43^ accompanied by attenuated degassing of the SO^9^. We interpret these shifts as reflecting increased SWW influence at ~ 44° S with a diminished influence south of ~ 55° S, over the SO, implying a northward shift of the SWW belt. Finally, recurrent negative glacier mass balance prior to ~ 12.5 ka was coeval with negative anomalies in SWW influence in northwestern Patagonia^40^, resumption of the atmCO_2_ rising trend^43^, and enhanced SO upwelling^9^. This correspondence suggests the occurrence of diminished SWW influence at ~ 44° S and stronger SWW influence south of ~ 51° S from a southward shift of the SWW belt during the YD.

The Cerro Riñón glacier chronology also shares similarities with mountain glacier chronologies from the mid and high northern latitudes^42^ (see Supplementary material Fig. S3). Those studies show glacier withdrawal from their LGM limits shortly after~ 18.5 ka^45,46^, broadly coinciding with the onset of T1, readvances between ~ 17 and 16 ka in multiple valleys of the European Alps^45–47^ and between ~ 16.5 and 15.7 ka in western North America^48^, broadly contemporaneous with the deposition of the CR1 moraine during HS1. A growing body of evidence indicates that glaciers readvanced between ~ 14.5 and 13.5 ka in the Norwegian Arctic^5^, between ~ 14.3 and 12.8 ka in east Greenland^49^, and between ~ 14.5 and 12.8 ka in western North America^50^, coeval with the formation of the CR2–CR4 moraines during the ACR. Subsequent moraine formation events between ~ 12.8 and 12.3 ka in east Greenland^51^, southern Alaska^6^, and Scotland^7^ occurred during the early YD, and several sites across the European Alps during the late YD^52–56^. These advances or standstills are indistinguishable in age with the CR5 moraine when considering dating uncertainties, and occurred just before widespread and profound ice recession to the valley headwalls. We acknowledge that significant glacial activity during T1 occurred within the YD and few dates overlapping with the ACR have been reported in the majority of northern hemisphere sites^42^. A large number of these glacier advances, however, culminated either early or late within the interval (see Supplementary material Fig. S3), indicating that glaciers were receding through much of the YD.

The pervasive interhemispheric synchrony in mountain glaciation during T1 lies in direct contrast to the largely antiphased polar ice core records (Fig. 3). Studies have attributed the severity of cooling in Greenland ice cores throughout HS1 and YD time to a seasonality switch related to episodes of extended sea ice cover in the North Atlantic region, skewing their isotopic records toward a predominantly winter temperature signal^57,58^. Mountain glacier fluctuations, in contrast, respond primarily to summer temperatures which remained comparatively warm during key episodes of T1 as reflected by modelling experiments complemented with multiproxy records from Europe^59^.

Our results and analysis show that the timing and structure of glacier fluctuations in the LPGV basin were coeval with climate events recorded in both the northern (i.e., HS1 and YD) and southern (i.e., ACR) hemispheres. From our interhemispheric comparison we interpret synchrony of mountain glaciers driven by a global climate signal during T1, challenging the expected antiphase behavior predicted from the bipolar seesaw paradigm. This finding favors an atmospheric mechanism for generating and globally propagating millennial-scale climate variability during T1. We conclude that high-to-middle latitude mountain glaciers fluctuated in phase during T1 partly in response to summer warming due to atmCO_2_ concentrations brought by changes in the SWW-SO coupled system^4^. Our findings further support recently hypothesized climate mechanism, dubbed the Zealandia Switch, which proposed that climate variability during T1 may have been triggered by orbitally-induced Southern Hemisphere warming and globally paced by changes on the austral atmospheric and oceanic circulation at millennial timescales^8^.

## Methods

### Geomorphological mapping

Detailed geomorphological mapping of the moraine limits in Lago Palena/General Vintter basin was conducted based on aerial photographs (GEOTEC 1:70,000—www.saf.cl), satellite imagery (Sentinel 2—https://scihub.copernicus.eu/) and digital elevation models (ALOS Palsar—https://search.asf.alaska.edu/). The preliminary map was checked during two field campaigns (March, September 2017 and January 2020).

### Rock samples collection

We collected 26 boulder samples for ^10^Be surface exposure dating from the Cerro Riñón moraines (CR complexes), in addition to one sample from a perched boulder (LV17-42) located immediately outboard of the outermost moraine limit, and 4 boulder samples from the innermost moraine ridge along the eastern shore of Lago Palena/General Vintter (i.e., PV6). We aimed for large boulders to avoid potential effects of post-depositional movements or posterior exhumation. Samples were taken from the upper ~ 4 cm of the boulder surfaces using a drill and explosive charges, avoiding areas exhibiting clear signs of erosion, such as spalling or flaking. Elevation and geographic coordinates of each rock sample were recorded with a handheld GPS unit (WGS84). We measured topographic shielding using a handheld compass and a clinometer. Moraine ages are interpreted as representing the culmination of moraine construction, and, thus, cold episodes, whereas the perched-boulder age provides a minimum age for ice retreat from the sampling site.

### Quartz separation and ^10^Be isolation

Initial crushing and sieving of the rock samples was carried out at the Pontificia Universidad Católica de Chile and subsequent quartz and beryllium extraction at the Cosmogenic Nuclide Laboratory at Lamont-Doherty Earth Observatory following the protocol outlined in^20,60^. ^10^Be/^9^Be ratios were measured at Lawrence Livermore National Laboratory Center for Accelerator Mass Spectrometry. Ratios were measured relative to the standard 07KNSTD with a ^10^Be/^9^Be ratio of 2.85 × 10^–12^ (^61^; ^10^Be half-life = 1.36 Myr.). Analytical raw data are available in the supplementary material S1. Given relatively recent improvements described in^20,60^, the average analytical uncertainty is ~ 2%, with almost half the analyses between 1.7% and 2.0%.

### ^10^Be surface exposure ages

^10^Be ages were calculated based on methods incorporated in the online exposure age calculator (v.3—https://hess.ess.washington.edu/math/v3/v3_age_in.html)^62^, considering the time-dependent Lal/Stone (Lm)^21,22^ scaling schemes and the regional Patagonian production rate (~ 50^o^ S)^20^, assuming zero erosion and a rock density of 2.65 g/cm^3^. We discuss ages based on Lm scaling scheme because it produces the ages that best fit with limiting radiocarbon data from the production-rate calibration site at Lago Argentino (~ 50° S)^20^. In addition, most of the Patagonia ^10^Be glacial chronologies were reported according to the same scaling scheme. We show in Table 1 that ^10^Be ages are statistically identical (i.e., accuracy) using other scaling methods. In the text, we report individual ^10^Be ages with 1σ analytical uncertainty and the standard error of the mean (SEM). Raw geographic and ^10^Be analytical data can be found in the Supplementary material Table S1. For comparison with other proxy records, mean moraine ages include the propagation of the analytical uncertainty and that of the local production rate (3%)^20^. Similarly, we recalculated available glacier ^10^Be chronologies from New Zealand^8^ by using the online exposure age calculator (v.3)^62^ incorporating a local production rate (~ 43.6° S)^63^. Recalculated ^10^Be ages are reported with the associated uncertainties (1σ), including a 2% propagation of the production rate error^63^ (See Supplementary material Tables S2, S3, S4 and S5). We note that Denton et al.^8^ used exposure age calculator v2.2^62^ based on^63^ and reported cosmogenic ages calibrated to the year A.D. 1950. Therefore, our recalculated ^10^Be ages differ from those in Denton et al.^8^ by 1–2%, which does not alter our interpretations.

## Supplementary Information

Supplementary Information.

## Data Availability

All data generated during the current study are included in this published article as a supplementary file.
