# Supplementary material for: Glacier fluctuations in the northern Patagonian Andes (44°S) imply wind-modulated interhemispheric in-phase climate shifts during Termination 1
Source: Sci Rep. 2022 Jun 27;12:10842. doi: 10.1038/s41598-022-14921-4 (PMC9237032; doi:10.1038/s41598-022-14921-4)

SUPPLEMENTARY MATERIAL for:

Glacier fluctuations in the northern Patagonian Andes (44°S) imply wind-modulated interhemispheric in-phase climate shifts during Termination 1

Rodrigo L. Soteres[1,2,*,] Esteban A. Sagredo[1,2,3], Michael R. Kaplan[4], Mateo A. Martini[1,2,5], Patricio I. Moreno[2,6,7,8]; Scott A. Reynhout[2,9], Roseanne Schwartz[4], Joerg M. Schaefer[4,10]

[1] *Instituto de Geografía, Pontificia Universidad Católica de Chile, Santiago, Chile*
[2] *Millennium Nucleus Paleoclimate, ANID - Millennium Science Initiative, Santiago, Chile*
[3] *Estación Patagonia de Investigaciones Interdisciplinarias UC, Pontificia Universidad Católica de Chile, Chile*
[4] *Lamont-Doherty Earth Observatory of Columbia University, Palisades, New York, USA*
[5] *Centro de Investigaciones en Ciencias de la Tierra (CONICET-UNC), Facultad de Ciencias Exactas, Físicas y Naturales, Universidad Nacional de Córdoba, Córdoba, Argentina*
[6] *Center for Climate Research and Resilience, Universidad de Chile, Santiago, Chile*
[7] *Institute of Ecology and Biodiversity, Universidad de Chile, Santiago, Chile*
[8] *Departamento de Ciencias Ecológicas, Universidad de Chile, Santiago, Chile*
[9] *Departamento de Geología, Universidad de Chile, Santiago, Chile*
[10] *Department of Earth and Environmental Sciences, Columbia University, New York, New York, USA*

*Corresponding author: Rodrigo L. Soteres (rlsoteres@uc.cl)
Instituto de Geografía, Pontificia Universidad Católica de Chile
Campus San Joaquín, Avda. Vicuña Mackenna 4860, Macul, Santiago de Chile

Table S1. Geographic and ^{10}Be analytical data sorted into moraine complexes of the Lago Palena/General Vintter in Patagonia with outliers (*). Boron-corrected ^{10}Be/^{9}Be values were measured against the 07KNSTD AMS standard with reported ^{10}Be/^{9}Be value of 2.85×10^{12} ([61]; ^{10}Be half-life = 1.36 Myr.). 1σ analytical or internal AMS uncertainties are shown. ^{10}Be concentrations are corrected for ^{10}Be in process blanks run with each sample batch. Density = 2.65 g/cm^{3}. Erosion correction = 0 mm/kyr. Carrier concentrations in ppm as follows, with the ^{10}Be/^{9}Be ratios measured in the process blanks in each sample batch: (a) 1031.92; 9.348 x 10^{-17}; (b) 1032; 2.51655 x 10^{-16}; (c) 1037.83; 6.21585 x 10^{-16}; (d) 1029.5; 9.94 x 10^{-16}; (e) 1030; 1.06362 x 10^{-15}.

ID Sample	Latitude (DD)	Longitude (DD)	Elev. (m. asl.)	Thickness (cm)	Shielding	Quartz (g)	^{9}Be Carrier (g)	^{10}Be/^{9}Be (10^{-14})	^{10}Be (10^{4} atoms/g)
**PV6**									
LVM17-19	-43.95039	-71.47274	966	1.5	0.99999	10.0587	0.1827 [d]	13.3693 ± 0.2753	16.7204 ± 0.3451
LVM17-20	-43.96147	-71.48231	986	1.46	0.99999	10.0695	0.1824 [d]	14.9934 ± 0.2778	18.7157 ± 0.3475
LVM17-21	-43.96136	-71.48222	987	2.12	0.99999	10.2666	0.1825 [d]	14.2141 ± 0.4076	17.4056 ± 0.4997
LVM17-23	-43.95932	-71.48016	988	1.73	0.99999	10.2027	0.1828 [d]	14.5319 ± 0.4113	17.9385 ± 0.5082
**CR1**									
LV17-15	-43.95631	-71.63477	989	0.83	0.99561	10.0593	0.1810 [b]	11.9671 ± 0.2223	14.7759 ± 0.2754
LV17-17	-43.95763	-71.63805	1028	0.92	0.99436	7.3368	0.1811 [b]	8.4723 ± 0.1780	14.3194 ± 0.3024
LV17-18	-43.95779	-71.63845	1033	0.66	0.98835	8.5175	0.1824 [a]	11.9671 ± 0.2223	14.8313 ± 0.2852
LV17-34	-43.95681	-71.63554	999	1.66	0.99431	10.0111	0.1817 [b]	10.0501 ± 0.1932	13.8238 ± 0.3937
LV17-35	-43.95674	-71.63504	991	2.03	0.99035	10.0145	0.1804 [b]	11.9671 ± 0.2223	13.4924 ± 0.3087
LV17-36	-43.95656	-71.63422	977	1.89	0.99531	10.0420	0.1810 [b]	10.8833 ± 0.2483	14.6171 ± 0.3836
LV17-41	-43.95595	-71.63452	993	1.44	0.99553	10.0267	0.1818 [c]	11.4006 ± 0.2171	14.1814 ± 0.2710
**CR2**									
LV17-20	-43.95951	-71.64014	1057	1.49	0.99168	9.8171	0.1806 [b]	9.5606 ± 0.2271	12.1003 ± 0.2883
LV17-21	-43.959	-71.6394	1051	0.99	0.99415	10.6714	0.1824 [b]	11.0940 ± 0.1887	13.0509 ± 0.2231
LV17-23	-43.95738	-71.63653	1016	1.47	0.99672	10.0129	0.1817 [c]	9.9408 ± 0.1855	12.3656 ± 0.2318
LV17-24	-43.95723	-71.63578	1007	1.25	0.99564	7.7729	0.1798 [b]	9.5606 ± 0.2271	12.3310 ± 0.2849
LV17-25	-43.95721	-71.63571	1006	0.96	0.99536	10.0773	0.1817 [c]	7.7533 ± 0.1782	11.9031 ± 0.2277
LV17-27	-43.9569	-71.63461	987	1.12	0.99631	10.5152	0.1811 [b]	9.7772 ± 0.1833	11.5858 ± 0.2183
**CR3**									
LV17-03	-43.95847	-71.63325	1000	0.98	0.99529	9.4877	0.1823 [a]	9.1395 ± 0.1725	12.1005 ± 0.2285
LV17-04	-43.95846	-71.63338	1003	1.71	0.99529	6.3327	0.1822 [a]	5.8934 ± 0.1295	11.6772 ± 0.2569
LV17-05	-43.95816	-71.6346	997	0.91	0.9947	10.0425	0.1818 [a]	9.1985 ± 0.2390	11.4743 ± 0.2982
LV17-06	-43.95891	-71.63708	1034	0.99	0.99307	10.0736	0.1811 [c]	9.5331 ± 0.1876	11.7448 ± 0.2321
LV17-07	-43.95936	-71.63822	1060	2.53	0.99282	7.1468	0.1801 [b]	6.9884 ± 0.1533	12.1040 ± 0.2675
LV17-08	-43.95942	-71.63835	1066	0.99	0.99467	5.4946	0.1823 [a]	5.3347 ± 0.1266	12.1869 ± 0.2895
LV17-12	-43.96037	-71.64056	1076	1.61	0.99337	10.3945	0.1822 [a]	10.0876 ± 0.1887	12.1851 ± 0.2281
**CR4**									
LV17-29	-43.9607	-71.63497	1017	0.83	0.98634	10.0145	0.1825 [a]	9.1901 ± 0.1884	11.5402 ± 0.2367
LV17-30*	-43.9607	-71.63545	1014	1.18	0.99355	11.0143	0.1818 [c]	8.3826 ± 0.1910	94.735 ± 0.2168
LV17-37	-43.96248	-71.63354	1030	1.36	0.98865	15.0622	0.1818 [c]	14.6167 ± 0.2916	12.1180 ± 0.2422
**CR5**									
LV17-38	-43.9689	-71.64705	1048	1	0.93103	14.0461	0.1821 [c]	12.9493 ± 0.2468	10.7591 ± 0.2056
LV17-39	-43.9688	-71.64677	1050	1.66	0.93104	12.0197	0.1817 [e]	10.4793 ± 0.1950	10.7913 ± 0.2020
**Perched boulder**									
LV17-42	-43.95883	-71.62435	962	1.56	0.99333	10.0267	0.1825 [c]	11.3490 ± 0.2922	14.1715 ± 0.3656

Table S2. Geographic and ^{10}Be analytical data sorted into moraine complexes of Rakaia Valley [31,32] in New Zealand with outliers (*). Final ^{10}Be ages were calculated in online exposure age calculator (v.3) [62] considering the time-dependent Lal/Stone (Lm) [21,22] scaling schemes and the regional production rate for New Zealand (~43.6° S) [63], assuming zero erosion and a rock density of 2.65 g/cm^3. We report individual ^{10}Be ages with 1σ analytical uncertainty.

ID Sample	Latitude (DD)	Longitude (DD)	Elev. (m. asl.)	Thickness (cm)	Shielding	^{10}Be (10^4 atoms/g)	^{10}Be age (yr)
**Big Ben**							
RAK-11-03	-43.387062	171.631847	733	2.20	0.99868	13.12 ± 0.28	17610 ± 380
RAK-11-04	-43.383056	171.628692	739	1.76	0.99948	13.01 ± 0.25	17330 ± 340
RAK-11-07	-43.380690	171.626590	719	1.53	1	13.17 ± 0.25	17830 ± 340
RAK-11-08*	-43.380540	171.626522	718	1.74	0.99718	12.42 ± 0.33	16870 ± 450
RAK-11-09	-43.377376	171.628256	746	1.99	0.99862	13.11 ± 0.37	17400 ± 500
RAK-11-10	-43.377154	171.628420	749	1.52	0.99897	13.10 ± 0.25	17290 ± 330
RAK-11-15	-43.369678	171.623158	648	1.83	0.99856	12.15 ± 0.23	17560 ± 330
RAK-11-16	-43.370223	171.622372	647	1.28	0.99896	12.09 ± 0.23	17350 ± 330
RAK-11-23*	-43.372421	171.618219	633	2.04	0.99865	12.72 ± 0.24	18520 ± 350
RAK-11-25	-43.374336	171.622657	651	1.65	0.99917	12.53 ± 0.31	17910 ± 450
**Prospect Hill**							
PH-08-01	-43.313559	171.110735	760	0.95	0.99819	12.13 ± 0.38	15820 ± 450
PH-08-02	-43.308400	171.122540	742	1.08	0.99497	11.81 ± 0.34	15720 ± 460
PH-08-03	-43.308260	171.122310	743	2.66	0.99849	12.34 ± 0.37	16520 ± 500
PH-08-04*	-43.305180	171.114400	884	3.17	0.99910	14.22 ± 0.36	17050 ± 430
PH-08-05	-43.305160	171.114400	885	1.15	0.99111	12.97 ± 0.35	15490 ± 420
PH-08-06	-43.305180	171.114400	885	1.03	0.99904	13.28 ± 0.30	15700 ± 360
PH-08-07*	-43.305220	171.113430	868	1.95	0.99839	11.62 ± 0.33	14050 ± 400
PH-08-08	-43.305470	171.113940	890	2.78	0.99726	13.67 ± 0.39	16360 ± 470
PH-08-09	-43.305980	171.113910	886	0.94	0.99648	13.50 ± 0.36	15950 ± 430
PH-08-10*	-43.306130	171.113390	872	1.00	0.99673	12.43 ± 0.37	14850 ± 450
PH-08-11	-43.311480	171.101290	821	1.24	0.99869	12.82 ± 0.30	15940 ± 380
**Reischek Knob Outer**							
RK-11-03	-43.291425	170.947971	1326	1.17	0.9759	15.76 ± 0.18	13570 ± 160
RK-11-07	-43.295769	170.948161	1448	2.08	0.9999	17.40 ± 0.19	13520 ± 150
RK-11-08	-43.295745	170.948151	1448	1.87	0.9888	17.67 ± 0.22	13740 ± 170
RK-11-10	-43.295343	170.947665	1442	2.66	0.9909	17.57 ± 0.21	13770 ± 170
**Reischek Knob Inner**							
RK-11-01	-43.29531	170.945962	1426	2.09	0.9939	16.35 ± 0.19	12930 ± 160
RK-11-02	-43.295231	170.945857	1425	2.30	0.9939	16.70 ± 0.27	13180 ± 210
RK-11-04	-43.291271	170.948296	1328	2.56	0.9752	15.00 ± 0.18	13120 ± 160
RK-11-05	-43.29082	170.949471	1300	1.08	0.9837	14.77 ± 0.17	12880 ± 150
RK-11-06	-43.295374	170.946695	1433	1.25	0.9891	16.01 ± 0.20	12560 ± 160
**Meins Knob**							
MK-11-01	-43.298686	170.917643	1299	1.30	0.9902	13.54 ± 0.19	11800 ± 170
MK-11-02	-43.297112	170.918453	1266	1.05	0.9905	13.52 ± 0.22	12050 ± 200
MK-11-03	-43.297064	170.918452	1265	1.41	0.9856	13.70 ± 0.19	12310 ± 170
MK-11-04	-43.296919	170.918755	1261	1.68	0.9902	13.38 ± 0.22	12070 ± 200
MK-11-05	-43.296912	170.918798	1261	1.63	0.9889	13.29 ± 0.25	12000 ± 230
MK-11-06	-43.296894	170.9188	1260	2.17	0.9879	13.40 ± 0.34	12150 ± 310
MK-11-07	-43.293861	170.920314	1270	1.57	0.9908	13.17 ± 0.25	11820 ± 230
MK-11-08	-43.293649	170.92051	1274	2.59	0.9939	13.20 ± 0.28	11840 ± 250

Table S3. Geographic and ^{10}Be analytical data sorted into moraine complexes of Lake Pukaki [33,34] in New Zealand with outliers (*). Final ^{10}Be ages were calculated in online exposure age calculator (v.3) [62] considering the time-dependent Lal/Stone (Lm) [21,22] scaling schemes and the regional production rate for New Zealand (~43.6^{o} S) [63], assuming zero erosion and a rock density of 2.65 g/cm^3. We report individual ^{10}Be ages with 1σ analytical uncertainty.

ID Sample	Latitude (DD)	Longitude (DD)	Elev. (m. asl.)	Thickness (cm)	Shielding	^{10}Be (10^4 atoms/g)	^{10}Be age (yr)
**Belt D**							
MB-07-27	-44.100514	170.246009	709	1.37	1	13.17 ± 0.38	17760 ± 510
MB-07-40	-44.137021	170.223387	648	1.99	1	12.44 ± 0.43	17640 ± 620
MB-07-41*	-44.133723	170.224243	654	1.95	1	15.17 ± 0.57	21390 ± 810
MB-07-42	-44.131335	170.225243	666	1.75	1	12.71 ± 0.41	17760 ± 580
MC-07-18	-44.074376	170.259044	746	2.97	0.996	13.35 ± 0.40	18460 ± 550
MC-07-19	-44.072628	170.258158	691	1.11	1	13.54 ± 0.40	18400 ± 550
MC-07-20	-44.071967	170.257979	691	2.82	1	12.45 ± 0.39	17300 ± 540
**Birch Hill Outer**							
BH-07-08	-43.854994	170.112755	584.8	1.51	0.997	9.08 ± 0.26	13750 ± 400
BH-07-09	-43.855111	170.11313	588.6	2.73	0.991	9.01 ± 0.26	13820 ± 400
**Birch Hill I**							
BH-06-02	-43.819065	170.106203	747.3	1.26	0.9994	10.65 ± 0.33	12780 ± 410
BH-06-03	-43.823093	170.105082	681.3	1.92	0.9992	9.57 ± 0.35	12180 ± 450
BH-06-04	-43.822836	170.105449	688.8	1.63	0.99	9.66 ± 0.17	12300 ± 220
BH-06-05	-43.81475	170.105398	765.6	2.25	0.998	10.34 ± 0.36	12270 ± 430
BH-06-06	-43.811233	170.104487	765.1	1.79	0.995	10.77 ± 0.33	12830 ± 390
BH-06-07	-43.808384	170.102947	772.9	1.15	0.995	10.62 ± 0.39	12470 ± 460
BH-07-10	-43.854687	170.11445	578.7	1.73	0.997	8.09 ± 0.22	12400 ± 340
BH-07-11*	-43.853697	170.114478	577.7	1.33	0.997	10.18 ± 1.01	15470 ± 1540
BH-07-12	-43.853602	170.11374	583.6	1.83	0.998	8.35 ± 0.25	12720 ± 380
BH-07-13	-43.837554	170.106321	679.1	1.86	0.993	9.35 ± 0.22	13190 ± 310
BH-07-14	-43.83554	170.10674	674.9	1.68	0.998	9.33 ± 0.21	13130 ± 300
BH-07-15	-43.835513	170.106733	674.9	2.01	0.998	9.14 ± 0.31	12910 ± 440
BH-07-16	-43.835497	170.106213	671.5	1.42	0.995	9.31 ± 0.26	13140 ± 370
BH-07-17	-43.833953	170.105455	674.3	1.35	0.994	8.99 ± 0.28	12700 ± 400
BH-07-18	-43.833708	170.105528	673.2	1.11	0.996	9.37 ± 0.27	13160 ± 380
**Birch Hill II**							
BH-06-09	-43.808516	170.114376	634.9	1.39	0.994	9.81 ± 0.55	12940 ± 730
BH-06-10	-43.812393	170.114462	607.6	1.94	0.995	9.48 ± 0.31	12850 ± 420
BH-06-11	-43.816018	170.11464	612.3	2.35	0.996	9.41 ± 0.29	12740 ± 390
BH-06-12	-43.816755	170.111584	629.6	1.62	0.995	9.91 ± 0.38	13130 ± 510
BH-06-13	-43.824295	170.113626	606.2	2.31	0.998	9.61 ± 0.31	13020 ± 420
KIWI 631	-43.823802	170.111548	605.5	3.1	0.995	9.58 ± 0.25	13110 ± 340
KIWI 632	-43.820842	170.114721	603.7	1.8	0.996	8.31 ± 0.18	12490 ± 270

Table S4. Geographic and ^{10}Be analytical data sorted into moraine complexes of Irishman basin [35] in New Zealand with outliers (*). Final ^{10}Be ages were calculated in online exposure age calculator (v.3) [62] considering the time-dependent Lal/Stone (Lm) [21,22] scaling schemes and the regional production rate for New Zealand (~43.6^{o} S) [63], assuming zero erosion and a rock density of 2.65 g/cm^3. We report individual ^{10}Be ages with 1σ analytical uncertainty.

ID Sample	Latitude (DD)	Longitude (DD)	Elev. (m. asl.)	Thickness (cm)	Shielding	^{10}Be (10^4 atoms/g)	^{10}Be age (yr)
**Irishman Basin Outer**							
IS-06-38	-43.9967	170.0358	1816	3.4	0.991	23.38 ± 0.55	13750 ± 330
IS-06-39	-43.9968	170.0358	1815	2.16	0.992	24.10 ± 0.57	14010 ± 330
IS-06-40	-43.9975	170.0357	1810	0.96	0.994	21.44 ± 0.51	12460 ± 300
IS-06-33	-43.9983	170.0401	1820	3.88	0.995	21.79 ± 0.47	12830 ± 280
IS-06-32	-43.9984	170.0401	1820	2.65	0.995	25.18 ± 0.68	14600 ± 390
IS-06-37	-43.9985	170.0377	1804	3.64	0.988	23.31 ± 0.55	13890 ± 330
IS-06-36	-43.9986	170.0372	1797	1.1	0.994	21.14 ± 0.42	12420 ± 250
IS-06-35	-43.9985	170.0383	1809	1.4	0.992	23.98 ± 0.56	13930 ± 330
IS-06-31	-43.9985	170.0394	1817	1.11	0.983	25.36 ± 0.60	14730 ± 350
**Irishman Basin Inner**							
IS-06-15	-43.9919	170.0491	2001	2.59	0.973	23.44 ± 0.54	12320 ± 290
IS-06-18	-43.9901	170.0513	2015	3.07	0.981	23.18 ± 0.68	12040 ± 350
IS-06-19	-43.9905	170.0505	2006	1.89	0.988	22.94 ± 0.48	11820 ± 250
IS-06-24	-43.9906	170.0475	1981	2.53	0.991	21.79 ± 0.47	11480 ± 250
IS-06-25	-43.9909	170.0468	1955	2.67	0.986	24.56 ± 0.53	13090 ± 290
IS-06-28	-43.9916	170.0443	1878	2.72	0.984	20.68 ± 0.59	11790 ± 340

Table S5. Geographic and ^{10}Be analytical data sorted into moraine complexes of Lake Ohau [36] in New Zealand with outliers (*). Final ^{10}Be ages were calculated in online exposure age calculator (v.3) [62] considering the time-dependent Lal/Stone (Lm) [21,22] scaling schemes and the regional production rate for New Zealand (~43.6^{o} S) [63], assuming zero erosion and a rock density of 2.65 g/cm^3. We report individual ^{10}Be ages with 1σ analytical uncertainty.

ID Sample	Latitude (DD)	Longitude (DD)	Elev. (m. asl.)	Thickness (cm)	Shielding	^{10}Be (10^4 atoms/g)	^{10}Be age (yr)
**Ohau IV**							
OH-06-08*	-44.28244	169.94186	557.3	2.21	0.999	12.65 ± 0.38	19490 ± 590
OH-06-10	-44.27704	169.98217	561.4	2.07	0.998	12.78 ± 0.40	17730 ± 560
OH-06-11	-44.27659	169.94047	559	1.45	0.99	12.85 ± 0.43	17950 ± 600
OH-06-13	-44.27492	169.93968	561.2	1.5	0.998	13.05 ± 0.34	18060 ± 470
OH-06-14	-44.27046	169.93488	564.1	2.07	0.992	12.26 ± 0.34	17120 ± 480
OH-06-15	-44.269896	169.9301	546.3	1.13	0.992	12.86 ± 0.34	18070 ± 480
OH-06-22	-44.29886	169.90032	583.5	2.81	0.999	13.19 ± 0.41	18020 ± 560
OH-06-23	-44.29853	169.90072	583.6	1.55	0.999	12.09 ± 0.37	18080 ± 560
OH-06-24	-44.2974	169.90386	578.3	1.65	0.995	13.42 ± 0.43	18270 ± 590
OH-06-26	-44.29836	169.90023	575.3	1.27	0.999	13.29 ± 0.39	18060 ± 530
OH-06-27	-44.29664	169.90778	579.2	3.33	0.999	13.55 ± 0.41	18700 ± 570
OH-06-31	-44.29227	169.93141	546.8	1.92	0.998	12. 85± 0.37	18060 ± 520
OH-06-84	-44.30176	169.87808	586.2	1.9	0.999	11.61 ± 0.36	17360 ± 540
OH-06-85	-44.30282	169.88131	582.9	2.09	0.999	13.41 ± 0.39	18200 ± 530
OH-06-86	-44.30214	169.89023	581	2.61	0.999	13.21 ± 0.39	18030 ± 540
OH-06-90	-44.30577	169.89184	580	2.51	0.999	12.52 ± 0.39	17090 ± 540
OH-06-92	-44.3067	169.88222	580	2.87	0.999	12.57 ± 0.38	17280 ± 520
OH-06-95	-44.30368	169.86948	591	1.68	0.999	13.57 ± 0.40	18280 ± 540
OH-06-96	-44.30026	169.86005	598	1	0.998	12.66 ± 0.47	16920 ± 630
**Ohau V**							
OH-06-25	-44.29694	169.90352	575.7	1.36	0.999	12.75 ± 0.39	17400 ± 530
OH-06-28	-44.29279	169.90362	554.8	1.94	0.998	12.00 ± 0.34	16710 ± 480
OH-06-29	-44.29291	169.90602	550.1	1.35	0.999	12.48 ± 0.39	17370 ± 540
OH-06-78	-44.28507	169.87905	540	2.15	0.998	12.48 ± 0.28	17650 ± 400
OH-06-79	-44.28508	169.87993	539.2	1.62	0.995	13.45 ± 0.41	19020 ± 580
OH-06-80	-44.28456	169.87991	539	1.78	0.998	12.52 ± 0.33	17610 ± 470
OH-06-88	-44.30005	169.88623	569.9	1.53	0.999	12.52 ± 0.30	17110 ± 410

Figure S1. Kernel density diagrams of ^{10}Be ages for the sequence of moraine ridges at Lago Palena/General Vintter and in the Cerro Riñón valley. Ages were calculated considering the in situ ^{10}Be production rate for Patagonia (~50°S) [20] and the Lal/Stone time-dependent scaling scheme (Lm) [21,22]. Red lines are Gaussian curves of individual ages. Thick black line is the sum of all samples in each moraine ridge. Blue line is the mean age. Yellow bar and green lines represent the 1σ and 2σ uncertainty ranges, respectively. In the text, aritmethic means are shown with 1σ and standard error of the mean (SEM) including 3% propagated production rate uncertainty. Chi square value is shown as Rχ2.

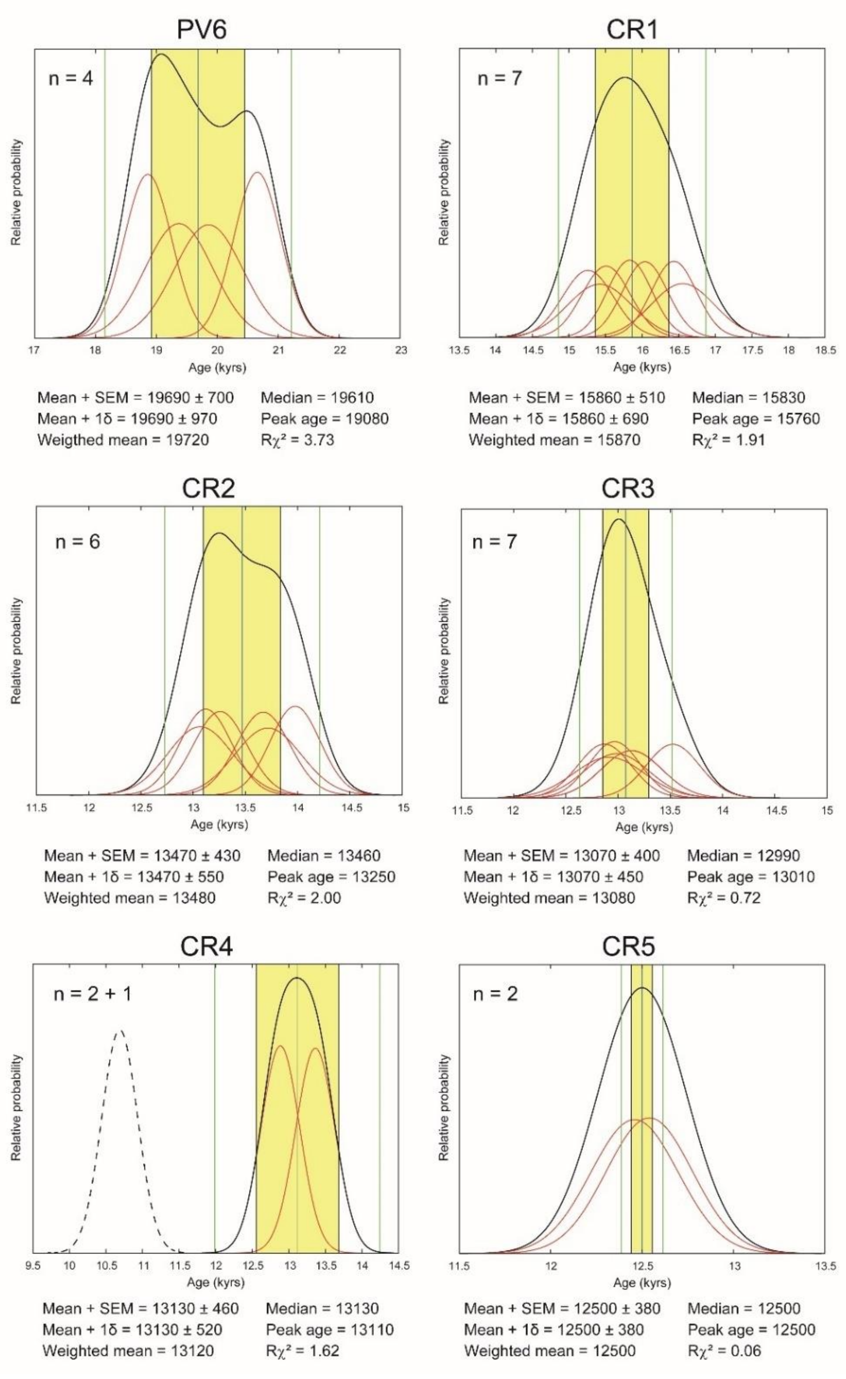

Figure S2. Overview of the main moraine complexes in the Cerro Riñón valley and examples of boulders sampled for surface exposure dating. a) and b) The CR1 moraine complex; c) and d) The CR2 moraine complex; e) and f) The CR3 moraine complex.

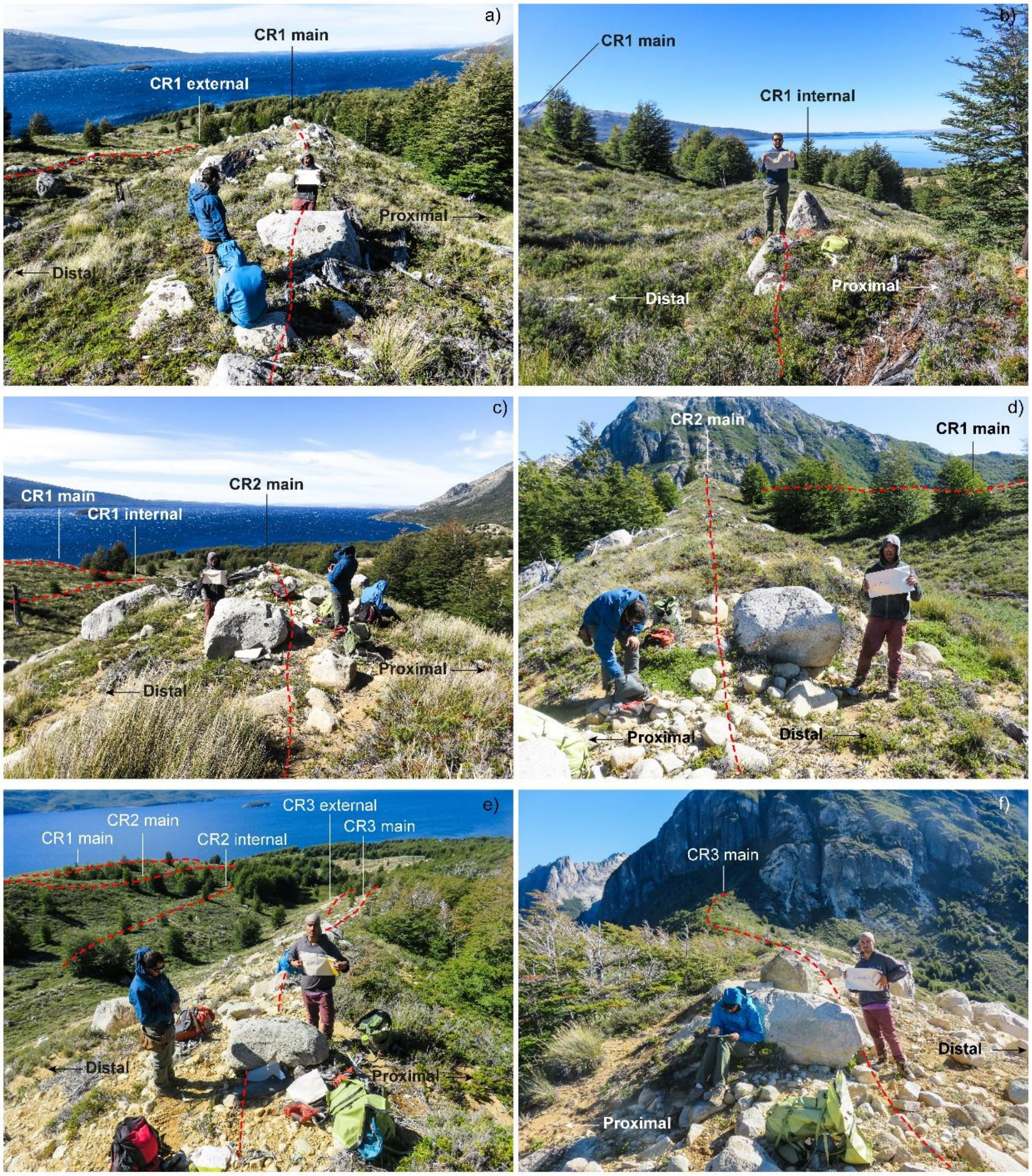

Figure S3: Globaly distributed glacier chronologies spanning T1. Circles correspond to mean moraine ^{10}Be ages accompanied by ±1σ range. Squares denote ^{10}Be ages from erratics and bedrock outcrops (±1σ). Triangles are bracketing radiocarbon ages with 2σ calibrated uncertainty. a) Glacier fluctuations in Patagonia [*this study,14,24,25,26,27,28,29]. b) Glacier fluctuations in the Southern Alps of New Zealand [31,32,33,34,35,36]. c) Glacier fluctuations in high latitudes of the Northern Hemisphere including Artic Norway [5], Alaska [6], Highland Scotland [7] and south Greenland [49,51]. d) Glacier fluctuations in Western United States [48,50]. e) Glacier fluctuations in the European Alps [45,46,47, 52,53,54,55,56]. f) CO_2 record from WAIS ice core [43]. Blue bars highlight cold/wet interval and yellow bars demote warm/dry periods inferred from palinological analyses [12,39].

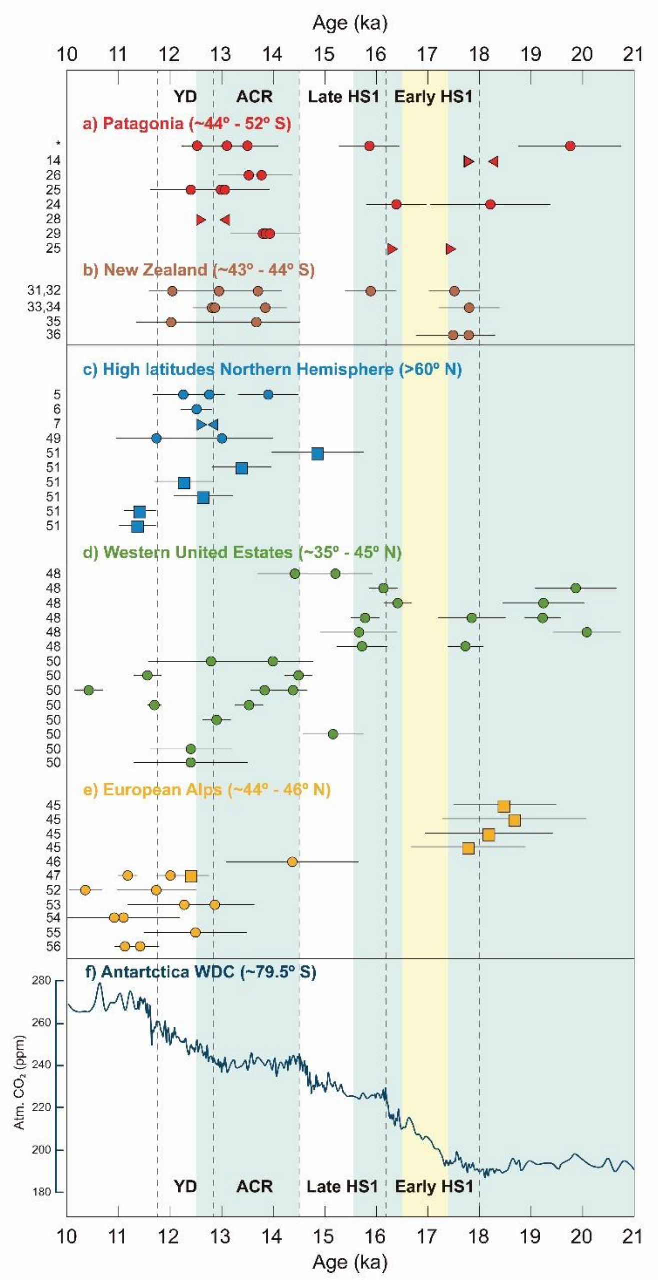

Supplement: Supplementary file 1 — Supplementary Information. [file 41598_2022_14921_MOESM1_ESM.pdf]
